# Development of a modified C-BARQ for evaluating behavior in working dogs

**DOI:** 10.3389/fvets.2024.1371630

**Published:** 2024-06-28

**Authors:** Elizabeth Hare, Jennifer Lynn Essler, Cynthia M. Otto, Dana Ebbecke, James A. Serpell

**Affiliations:** ^1^School of Veterinary Medicine, University of Pennsylvania, Philadelphia, PA, United States; ^2^Dog Genetics LLC, Astoria, NY, United States; ^3^College of Agriculture and Technology, SUNY Cobleskill, Cobleskill, NY, United States

**Keywords:** dogs, odor detection, behavior, assessment, questionnaire, C-BARQ

## Abstract

**Introduction:**

Current high demand for effective odor detection dogs calls for the development of reliable methods for measuring performance-related behavioral phenotypes in these highly specialized working animals. The Canine Behavioral Assessment & Research Questionnaire (C-BARQ) is a widely used behavioral assessment tool among working dog organizations with a demonstrated ability to predict success/failure of dogs in training. However, this instrument was developed originally to study the prevalence of behavior problems in the pet dog population, and it therefore lacks the capacity to measure specific behavioral propensities that may also be important predictors of working dog success. The current paper examines the factor structure, internal reliability, and content validity of a modified version of the C-BARQ designed to evaluate four new domains of canine behavior in addition to those encompassed by the original C-BARQ. These domains, labeled *Playfulness*, *Impulsivity*, *Distractibility*, and *Basophobia* (fear of falling), respectively, describe aspects of canine behavior or temperament which are believed to contribute substantially to working dog performance.

**Methods:**

Exploratory factor analysis (EFA) of owner/handler questionnaire responses based on a sample of 1,117 working odor detection dogs.

**Results:**

A total of 15 factors were extracted by EFA, 10 of which correspond to original C-BARQ factors. The remaining 5 comprise the four new domains– *Playfulness*, *Impulsivity*, *Distractibility*, and *Basophobia*– as well as a fifth new factor labeled *Food focus*.

**Discussion:**

The resulting Working Dog Canine Behavioral Assessment & Research Questionnaire (WDC-BARQ) successfully expands the measurement capacities of the original C-BARQ to include dimensions of behavior/temperament of particular relevance to many working dog populations.

## 1 Introduction

Detection dogs are commonly used to find various substances based on odor, many of which they can find more efficiently or more quickly than humans. The fields in which detection dogs are deployed are numerous and growing, including explosives detection (1, 2), narcotics detection (3), medical detection (4–7), live human search and rescue (SAR) (8–10), human remains detection (11, 12), and conservation detection (13–17). With the increased use of detection dogs, the number of suitable candidates available has not met the demand (18, 19). One of the most critical reasons for this shortage is the necessary high standards for these working dogs, which results in a high release rate for detection dogs in training. Ultimately, any dog released from training means the loss of those man-hours and monetary costs that are not recuperated—resources that could have been allocated to another dog that may have been successful. Thus, there has been a push to improve selection of detection dogs (3, 20–25), even as early as 3 months of age (20).

Selection of better detection dogs earlier, and more efficiently, requires defining an ideal detection dog phenotype: a specification of which behavioral traits are valuable and which are detrimental. There are difficulties in trying to define these behavioral traits (21). First, both between-breed and within-breed variation are important (22–24). Sometimes assessments of breeds do not match industry preferences. For example, when handlers evaluated their own dogs on a number of traits (e.g., motivation and distraction), Border Collies and English Springer Spaniels were found to be more suitable for drug and explosives detection work than Labrador Retrievers, despite Labrador Retrievers being historically considerably more popular (though Spaniels are growing in popularity in the United States) (23). Second, there are many terms that are commonly used in the field to describe detection dogs which are difficult to define and almost impossible to measure. “Drive,” for example, is often used to describe detection dogs, and was defined by the National Institute of Standards and Technology (NIST) and published as an American Standards Board technical report operationally as ‘a willingness, vigor, or enthusiasm to engage in certain behavior, contexts, or situations.’ [Crime Scene/Death Investigation – Dogs and Sensors – Terms and Definitions | American Academy of Forensic Sciences^1^]. Thus, dogs with ‘high drive’ are desirable for detection work, while dogs with ‘low drive’ are not. Though it can be relatively easy to compare two dogs on some spectrum of ‘drive,’ it is difficult to quantify the trait in an individual dog. Finally, in contrast to the operational setting where career longevity is a vital parameter, the determination of a ‘successful’ detection dog in research studies is typically based on passing an initial certification or ‘graduation’ from its specific program, while later or on-going ‘success’ in the dog’s working career is only rarely reported (25, 26). This limited perspective impedes assessment of specific traits or behaviors associated with long-term success. One study found that 17% of working guide dogs that passed their training program were withdrawn for behavioral issues after being placed in service (27); if the researchers had been assessing success merely as having passed the training program, their results would have been significantly different. Thus, investigation of detection dog performance beyond initial training and certification is necessary to truly define the traits associated with success (20).

Currently, most working dog programs apply their own internal assessments of their dogs, usually within the first 2 years of life. For example, when the United States Transportation Security Administration ran their breeding program, they used their Standardized Behavioral Tests to evaluate puppies at 3, 6, 9, and 12 months of age (28). These tests assessed environmental soundness such as auditory sensitivity, as well as their propensity to chase and retrieve a toy. Individual raters scored the dogs during the tasks on five-point Likert scales based on the observed behaviors. Research on these live assessments compared to coding, where individuals watch recorded videos of the tasks and score specific behaviors to assess the dogs, found that both methods were statistically similar in their ability to classify dogs based on training outcomes (29). However, the time required to rate the dogs during live tasks, which included the task time, was significantly lower than the time necessary to record and code behavior from videos. In another study, a population of German Shepherd Dogs and Labrador Retrievers at the Swedish Dog Training Centre were scored for behavioral characteristics across multiple tasks by one rater on multiple Likert scales, which resulted in the ability to successfully select dogs for working careers (24). This is a common trend in the working dog field, where working dog kennels assess their own dogs based on their own tests and scoring systems, making it difficult to compare or combine results across different working dog populations. Without these comparisons, it is impossible to select reliably for behavioral traits that could result in increased breeding success, selection, and performance of working dogs (21).

Phenotyping through owner or handler questionnaires is becoming increasingly popular in investigations into dog behavior, as they allow the assessment of large numbers of dogs for minimal cost and effort and are more likely to record relatively uncommon behavioral responses that would likely be missed in single tests or observation periods or using simple personality descriptors. Probably the most frequently used of these questionnaires is the C-BARQ (Canine Behavioral Assessment and Research Questionnaire), which has become one of the industry standards for measuring dog temperament and behavior in both non-working (30–32) and working dog populations (10, 33, 34). The questionnaire comprises 14 behavioral subscales extracted by Exploratory Factor Analysis including different types of aggression and fearfulness/anxiety, trainability, excitability, predatory chasing, and attachment/attention-seeking, and a further 27 miscellaneous items ranging from coprophagia to stereotypic spinning/tail-chasing. In one study conducted in 5 different guide and service dog organizations, volunteer puppy-raisers completed C-BARQs when dogs were 6 and 12 months of age (33). The results from these surveys were used to assess whether a dog’s success (being placed with a handler or selected as a breeder) or released from the program for behavioral reasons could be predicted based on its earlier C-BARQ scores. Guide/service dogs that successfully completed their training obtained more favorable scores on 27 out of 36 C-BARQ behaviors and ‘pulling excessively hard on the leash’ was the most highly predictive trait for failure. Logistic regression models also indicated that the overall C-BARQ evaluations were able to discriminate between successful and unsuccessful dogs significantly above chance levels (areas under the ROC curves 0.64–0.72). A subsequent study within a single service dog organization obtained similar results but also determined that C-BARQ evaluations were more accurate at identifying dogs with the lowest probabilities of being successful (85–92%) compared with the most successful dogs (62–72%) (35). In a study of Search and Rescue (SAR) dogs, handlers filled out C-BARQs and the results were compared with those from a different population of pet dogs belonging to the same breeds (10). The survey results found significant behavioral differences between the two populations. For example, SAR dogs obtained higher scores for the trait of trainability, and lower scores for aggression and fear. These findings are similar to those obtained in a study of German Shepherd Dogs from the Swedish Military Working Dog Program that assessed dogs using the C-BARQ as well as a standardized behavioral test of suitability for further training (34). In this case, dogs that scored higher on trainability were more successful and dogs that scored higher on different levels of fear were less successful. However, dogs that scored higher on traits that would typically be considered negative in a pet dog—for example ‘hyperactive/restlessness, difficulty in settling down’—were associated with greater success in the standardized test in the working dogs. Such findings underline the fact that the desirability of different behavioral traits likely varies according to dogs’ functional roles, and that a questionnaire instrument originally developed to evaluate companion dogs (e.g., C-BARQ) may fail to assess some of the behavioral characteristics that contribute to the success of working dogs.

The primary goal of the current study was therefore to develop and validate a modified version of the C-BARQ including a novel set of questionnaire items designed to measure behavioral traits that are widely considered to be important to the success of working dogs. For example, working dogs tend to display higher rates of playfulness than pet dogs (36) and many working dog programs make use of a dog’s motivation to engage in object-play (e.g., overall interest in playing with toys/objects and motivation to search for and retrieve toys/objects) as an effective reward-based training paradigm (20, 28, 36, 37). Four new questionnaire items designed to measure aspects of this play motivation were therefore added to the WDC-BARQ prototype. Similarly, the ability to control or inhibit immediate impulses and cope with frustration—variously termed ‘self-control,’ ‘inhibitory control,’ ‘impulse control,’ and ‘frustration tolerance’—is widely regarded as a desirable trait in both working and companion dogs (38, 39). Four new questionnaire items addressing aspects of this trait were therefore added to the questionnaire prototype. Behavioral syndromes analogous to attention-deficit/hyperactivity disorder (ADHD) in humans have also been identified in dogs (40–42) and are characterized by symptoms of inattention, distractibility, hyperactivity, and impulsivity—traits which are generally considered undesirable in most working dogs (43–45). Six new questionnaire items were included in the prototype WDC-BARQ focusing mainly on inattention/distractibility (since hyperactivity and impulsivity are addressed by other new or existing items in the questionnaire). Finally, the majority of working dogs are required to work effectively over difficult substrates, including staircases (open and closed risers), slippery floors, and ‘visual cliff’ effects such as sidewalk grates. Dogs that consistently balk at such substrates are generally released from further training (46, 47). Since the original C-BARQ already contains a single questionnaire item pertaining to a dog’s tendency to be nervous or frightened on stairs, two additional items were added to the new survey to gauge responses of this type.

Finally, a secondary goal of the present analysis was to determine the extent to which the original factor structure of C-BARQ would be disrupted by the addition of questionnaire items addressing these new behavioral domains.

## 2 Methods

### 2.1 Ethical review

This research was pre-reviewed by the University of Pennsylvania Office of the Institutional Review Board and determined not to require IRB review.

### 2.2 Subject recruitment and data collection

We recruited owners/handlers of odor detection dogs from working dog organizations and by social media to complete the prototype, online, Working Dog C-BARQ (details below). Working dog owners/handlers were recruited via various social media platforms including Facebook, Instagram, LinkedIn, and X (formerly Twitter). Owners were also recruited using business cards with QR codes linking to the survey, and through word-of-mouth from representatives of the Penn Vet Working Dog Center during relevant conferences and training events.

Data collection occurred in two contiguous phases because questions concerning sterilization status and the C-BARQ ‘chasing items’ were inadvertently excluded from the first phase. Data from phases 1 and 2 were analyzed together because the sample sizes for the two subgroups were inadequate for separate analyses. There were 734 dogs in Phase 1 and 411 dogs in Phase 2, resulting in a total of 1,145 dogs. After matching for dog name and handler email address, we found 28 duplicated dogs. In most cases, we retained only the latest survey for each duplicated dog. In the case where two surveys were completed for the same dog on the same day, the one with the fewest missing values was retained. After removing duplicates, 1,117 dogs remained in the analysis.

### 2.3 Sample demographics

Demographic questions in the survey included date of birth, country, breed, sex, and sterilization status. Breeds were assigned to breed groups according to the American Kennel Club (AKC)’s breed groupings. For breeds not listed by AKC, we assigned dogs to groups based on breed history and purpose. We removed the chasing questions (MISC72, MISC73, and MISC74) from the Phase 2 data because they were missing in the Phase 1 version of the questionnaire.

### 2.4 C-BARQ modifications for working dogs

#### 2.4.1 Original and modified C-BARQ items

High priority was given to reducing the total number of questionnaire items in the working dog version of the C-BARQ to reduce the survey burden on dog trainers and handlers. The C-BARQ was developed originally to assess the prevalence and severity of behavior problems in the pet dog population (35). Consequently, more than half of its original 100 items address various forms of aggression and fear/anxiety. Since most working dogs are pre-selected to show low levels of aggression and fear, the number of items focusing on these domains of behavior was reduced substantially from 26 to 12 for aggression, and from 26 to 14 for fear/anxiety items (including separation-related anxiety). Items in the factors labeled ‘Excitability’ and ‘Attachment/Attention-seeking’ were both reduced from 6 to 4, and the number of Miscellaneous items was decreased from 27 to 16. The ‘Energy’ and ‘Trainability’ factors retained their original number of items (2 & 8, respectively), but the original wording of the ‘Trainability’ items was changed to avoid the need to reverse the scores for some items. Decisions regarding which items to retain or discard were based on how strongly the items loaded on their original factors or subscales and on how likely working dog handlers/trainers would be able to respond accurately to the items. Some related items that were originally separate were also merged in the new version, and several items were rephrased to make them more applicable to working dog contexts and scenarios (see Supplementary Table 1).

#### 2.4.2 New working dogs C-BARQ items

*Playfulness:* Four new questionnaire items designed to measure aspects of this play motivation were added to the WDC-BARQ prototype: the dog’s enthusiasm for engaging in play with new/unfamiliar people, its level of focus on play objects, its tendency to initiate play and retrieve thrown toys/objects, and its persistence in searching for thrown or hidden toys/objects. *Impulsivity:* Four new questionnaire items addressing aspects of impulsivity were added to the WDC-BARQ prototype, including items aimed at assessing a dog’s overall impulsiveness, its impatience/frustration across a wide range of situations, its persistence in pursuing a desired goal, and its tendency to display stereotypic/repetitive behavior when prevented from accessing something it wants. *Distractibility:* Six new questionnaire items were included in the prototype WDC-BARQ focusing mainly on inattention /distractibility (since hyperactivity and impulsivity are addressed by other new or existing items in the questionnaire). *Basophobia (fear of falling):* The original C-BARQ contains a single questionnaire item pertaining to a dog’s tendency to be nervous or frightened on stairs, so two additional items were added to the new survey to gauge responses to slippery floors and sidewalk grates. All original C-BARQ and new or modified WDC-BARQ survey items are provided in full in Supplementary Table 1.

### 2.5 Statistical analysis

R (48) was used for all analyses. The code for this project is located in a GitHub repository at: https://github.com/LizHareDogs/detectorCBARQ.

Several methods were used to characterize the C-BARQ items to evaluate their suitability for data reduction through factor analysis, and to decide how to model the items’ distributions. To evaluate the statistical distribution of C-BARQ items, we calculated the number of missing observations, mean, standard deviation, minimum, maximum, and Shapiro–Wilk test for normality for each item. The ‘skew’ and ‘KURTOSI’ functions from the ‘psych’ R package (49) were used to calculate the skewness and kurtosis, respectively. There were no invalid data points (item values less than 0 or greater than 4). Bar charts were produced to visualize the distribution of item responses.

Two methods were used to test the data set containing all the items together for factorability. Bartlett’s Test of Sphericity was used to test whether the covariances between the items were significantly different from zero, which would indicate the presence of measurable variation for computation of factors. The Kaiser-Meyer-Olkin (KMO) criterion measures the degree to which each item is correlated with all the other items and is an additional method of ascertaining that the items have sufficient covariance for factoring.

Polychoric correlations between all items were computed for raw (Supplementary Data Sheet 1) and imputed (Supplementary Data Sheet 2) data sets using the ‘polychoric’ function in the ‘psych’ package (49). We set options for smoothing to ensure the result was a positive definite matrix, which is required for subsequent steps and for correction (0.01) to avoid computational problems with the many item values that were zero. We determined the range of correlations between items to ensure that there was sufficient variation and that none of the items had correlations over 0.90.

#### 2.5.1 Exploratory factor analyses

Three sets of exploratory factor analyses were performed. Set 1 used data from all items and contained analyses with 11–18 factors. For Set 2, we eliminated unsuitable items based on the results of Set 1. Set 3 contained the best model from Set 2 after removal of an item with low communality that was theoretically incompatible with the other items in the factor.

The five-category item responses, which represented either the frequency or intensity of each behavior, were assumed to be ordered categorical variables rather than continuous variables because of their statistical distributions. The significant (*p* < 0.001) Shapiro–Wilk test results indicated that the distributions were different from normal. Items with skewness greater than 2.0 or kurtosis greater than 7.0 should not be considered normally distributed for the purposes of factor analysis (50). In this survey, 17 items would have been disqualified due to high skewness or kurtosis if we had chosen to analyze the items as continuous normal rather than ordered categorical data. For this type of data, models that assume items are normally distributed could provide imprecise and spurious findings (50, 51).

Missing values were imputed because removing all records with any missing values would have left us with a data set too small to analyze. Imputation is recommended over removal or the use of mean or median values (52–54). Missing questionnaire items were replaced with values from a multiple imputation process for ordered categorical values implemented in the ‘imputeMCA’ function in the ‘missMDA’ package (55, 56).

Two methods were used to estimate the number of latent factors present in this data set: parallel analysis (PA) and minimum average partial (MAP). PA, implemented in the ‘fa.parallel’ function in the ‘psych’ package (49), compares eigenvalues between the data under analysis and random data and only factors with eigenvalues above the mean of those from the random data are retained, and is recommended for categorical data and polychoric correlations (50, 57, 58). The second method, MAP, is the number of factors that minimizes the average squared partial correlation and may be more accurate with polychoric correlations than with Pearson correlations (50). The MAP is calculated using the ‘VSS’ function in the ‘psych’ package with options for parallel analysis solution for the model, oblimin rotation, smoothing, correction for zero scores, polychoric correlation, and maximum iterations = 100,000.

In Set 1, an exploratory factor analysis model was calculated for each possible number of factors between 11 and 18 to compare model fit, item communalities and loadings, and the behavioral similarity of items within factors. We used the ‘fa’ function from the ‘psych’ package (49) with the imputed data set as input. The options for all factor analyses in Sets 1–3 were principal axis (PA) solution method, oblimin rotation, smoothing, polychoric correlation, and correction for zero values. Either weighted least squares (WLS) or PA solution methods are recommended for models with ordinal data (50, 58, 59). PA is also recommended when there could be a small number of items in some of the factors (60). We were unable to use WLS because of computational errors estimating the weights, so only PA was used. The purpose of rotation is to make factors more interpretable by rotating the axes representing the factors so that the factors are graphically closer to the items comprising them (52, 58). Oblique rotation methods such as oblimin allow non-zero correlations between factors (58) as we expect for groups of factors such as owner, stranger, and dog-directed aggression. Smoothing and setting correction to 0.01 are options to ensure correlation matrices are positive definite to avoid computational errors (49, 61). Using a polychoric correlation to fit ordinal items rather than a correlation for normally-distributed continuous variables reduces bias in extracting factors (50, 62–64).

To assess model fit, the chi-square test and the Tucker-Lewis Index (TLI) of factoring reliability were used. The chi-square test can be used for ordinal models (50) to ask whether the model is consistent with the polychoric correlation matrix (49) and is frequently used for all factor analysis computation methods. The TLI has been found to be one of the best measures of model fit (65). The chi-square fit and Tucker-Lewis Index of Factoring Reliability (TLI), two measures of the fit of the factor model, as well as communalities and loadings for each factor, were extracted from the analysis output for each model. Examination of the output of these models revealed some low communalities (<0.40) and low factor loadings (<0.32). Some items had >88% zero (“never” or “none”) responses which probably caused errors calculating reliabilities of some models.

A second set of models under the same analysis conditions with edited data sets were fit for models with 12 to 18 factors. Separate re-imputed data sets were input for each number of factors, depending on the results of the first set of analyses. Items were removed if (1) their communalities were <0.40, (2) their loadings were <0.32, and/or (3) >88% of responses were zero (‘never’ or ‘none’).

The data were analyzed in three stages. Set 1 examined the full imputed data set with number of factors set to 11 through 18. Based on the results of these models, data sets were created for Set 2. For each potential number of factors, the data set was edited to contain only items with communalities, loadings, and response frequencies meeting our criteria for Set 1 models with the same number of factors. After analyzing Set 2, the 15-factor model was selected based on the behavioral relationships between the items in each factor. One item (MISC69: “Chases/follows shadows, light spots, etc.”) that grouped incongruously with the Basophobia factor was eliminated because it had a low communality (0.20) and grouped with items MISC54, MISC55, and MISC56, which capture nervousness on, or reluctance to cross, some types of stairs, surfaces, or floors, respectively.

The recommended number of factors by PA and MAP are shown in Supplementary Table 2. To decide how many factors to retain, we considered the number of factors suggested by statistical procedures, the theoretical relationships between behaviors, and results of previous research (56). We rejected models with fewer than 15 factors because they formed factors that contained incongruous items. Supplementary Table 3 describes the items that were removed after the first set of factor analyses.

Two measures of reliability were calculated for each model in Sets 2 and 3: Cronbach’s alpha, which measures the agreement between the items within each factor, and omega, which is a reliability measure based on the entire data set. Cronbach’s alpha was computed with the ‘scores’ function in the ‘psych’ package (49). The inputs to this function are the list of keys for the factors of the specific model and the original imputed data set. To calculate omegas, we first computed the Schmid-Leiman transformation on the results of the factor analysis using the ‘SL’ function from the ‘EFAtools’ package (66) with options for parallel analysis and type ‘psych.’ The Schmid-Leiman transformation results were input to the ‘OMEGA’ function from the same package with the option for ‘psych’ type. Omega is calculated with the assumption that there is a general factor, g, that explains the proportion of variance that all the items in the model share. Total omega is the total score variance for the factor. Hierarchical omega is the variance due to the general factor, g. Subscale omega is the variance attributable to the factor (67).

## 3 Results

### 3.1 Demographics

There were 1,117 dogs in this study, with 686 from Phase 1 and 431 from Phase 2. The mean age at the time of the survey was 4.82 years (SD = 3.32). The sample was 56% male and 44% female. The dogs were 79% “purebred,” 8% “crossbreed,” and 6% “mixed breed” dogs. Most of the dogs were from Herding (51%) and Sporting (43%) Groups (Tables 1, 2). The purebred part of the population was diverse, with 105 breeds represented (Supplementary Table 4), although the majority of the sample comprised just six breeds or breed-types (Supplementary Table 5). Most dogs (87%) lived in the United States, with small numbers of dogs from 26 other countries.

**Table 1:** Frequencies of breed groups of participating detection dogs.

Breed group	*N* (%)
Herding	449 (50.7%)
Hound	12 (1.4%)
Non-sporting	9 (1.0%)
Sporting	382 (43.1%)
Terrier	12 (1.4%)
Toy	1 (0.1%)
Working	21 (2.4%)
Unknown	231

**Table 2:** Frequencies of odor detection types among participants’ dogs.

Type of trained odor	*N* (%)
Accelerant	17 (1.5%)
Contraband	15 (1.3%)
Endangered species	26 (2.3%)
Explosives	112 (10.0%)
Human remains	372 (33.3%)
Invasive species	18 (1.6%)
Live human air scent	322 (28.8%)
Live human tracking	234 (20.9%)
Medical detection	21 (1.9%)
Narcotics	146 (13.1%)
Pests	29 (2.6%)
Other	247 (22.1%)

Supplementary Table 6 contains the number of missing observations, mean, standard deviation, maximum, minimum, skewness, kurtosis, Shapiro–Wilk statistic, and Shapiro–Wilk *p*-value for each item. Supplementary Data Sheet 1 contains the polychoric correlation matrix of the raw data, and Supplementary Data Sheet 2 contains the polychoric correlation matrix after imputation of missing values.

We used Bartlett’s Test of Sphericity and the Kiser-Meyer-Olkin (KMO) Criterion to assess whether the raw and imputed data sets contained enough covariance between items for factoring. The Bartlett chi-square was 72,401 (2,485 DF, *p* < 0.01) for the raw data and 66,347 (2,485 DF, *p* < 0.01) for the imputed data. The significant results indicate that the covariances between the items are above zero. The KMO was 0.857 for the raw data and 0.861 for the imputed data set. This statistic ranges from 0 to 1 and compares the variance shared by all items with the variance due to pairs of items. The recommended minimum acceptable values vary from 0.5 to 0.7 (50), suggesting that our data had sufficient covariance to be factorable.

### 3.2 Factor analysis

The data were analyzed in three stages. Set 1 examined the full imputed data set with number of factors set to 11 through 18. Based on the results of these models we created data sets for Set 2. For each potential number of factors, we edited the data set to contain only items with communalities, loadings, and response frequencies meeting our criteria for Set 1 models with the same number of factors. After analyzing Set 2, we selected the 15-factor model based on the behavioral relationships between the items in each factor and removed an item that grouped incongruously with the *Basophobia factor*. The recommended number of factors by PA and MAP are shown in Supplementary Table 2. Supplementary Table 3 describes the items that were removed after the first set of factor analyses.

For the analysis in Set 3-Table 3, each survey item is shown with its corresponding factor, communality, highest loading, and a verbal description of the item. All communalities were at least 0.40, and their range was 0.40–0.90. The item loadings varied from 0.39 to 0.88. Factors were assigned two, three, four, or six items. There were two items for dog-directed aggression, food focus, energy, and attachment/attention-seeking; three items for *basophobia*, *separation-related behavior*, *touch sensitivity*, and *impulsivity*; four items for *stranger-directed fear*, *playfulness*, *dog-directed aggression*, *excitability*, *trainability*, and *stranger-directed aggression*; and six items for *distractibility*.

**Table 3:** Results of Set 3 factor analysis: each survey item shown with its corresponding factor, label, communality, highest loading, and verbal description.

Factor	Item #	Factor label	Communality	Maximum loading	Description
1	FEAR21	Stranger-directed fear	0.84	0.83	Displays fear when an unfamiliar person approaches the dog when s/he is away from his/her normal home environment or kennel
1	FEAR23	Stranger-directed fear	0.8	0.76	Displays fear when an unfamiliar person visits your home or approaches the dog when in his/her home kennel
1	FEAR24	Stranger-directed fear	0.9	0.87	Displays fear when an unfamiliar person tries to touch or pet the dog
1	FEAR28	Stranger-directed fear	0.63	0.45	Displays fear when first exposed to unfamiliar situations (e.g., novel environments, first visit to the veterinarian, etc.)
2	PLAY43	Playfulness	0.55	0.51	Eagerly engages in play with new/unfamiliar people
2	PLAY44	Playfulness	0.84	0.88	Highly toy focused; attention riveted on tug toy/balls when these are held by handler or other persons
2	PLAY45	Playfulness	0.77	0.84	Eagerly initiates play sessions; brings objects/toys to you/the handler and retrieves them when thrown
2	PLAY46	Playfulness	0.72	0.8	Hunts persistently for thrown or hidden toys/objects, not easily distracted from this task
3	MISC62	Distractibility	0.45	0.39	Becomes highly excited and/or distracted when encountering unfamiliar dogs
3	MISC64	Distractibility	0.6	0.79	When working, is easily distracted or preoccupied by odors/engages in persistent sniffing of ground or objects
3	MISC65	Distractibility	0.65	0.75	Has difficulty shifting attention away from interesting or distracting stimuli (e.g., other dogs, odors, people, small animals, etc.)
3	MISC66	Distractibility	0.69	0.62	Is distracted or nervous in new, unfamiliar environments, has difficulty maintaining focus on work
3	MISC67	Distractibility	0.59	0.57	Is slow to recover after being distracted, startled, or frightened/takes a long time to resume work
3	TRAIN07	Distractibility	0.62	0.66	Unfocused; is easily distracted by interesting sights, sounds or smells
4	AGG16	Dog aggression	0.76	0.5	Barks, growls, attempts to bite when approached directly by an unfamiliar dog while being walked/exercised on a leash
4	AGG18	Dog aggression	0.75	0.52	Barks, growls, attempts to bite when barked, growled, or lunged at by another dog
4	AGG19	Dog aggression	0.79	0.87	Acts aggressively toward other familiar dogs
4	AGG20	Dog aggression	0.52	0.68	Barks, growls, attempts to bite when approached while playing with/chewing a favorite toy, bone, object, etc. by another familiar dog
5	MISC54	Basophobia	0.64	0.68	Reluctant to/nervous about crossing grates or other unfamiliar surfaces
5	MISC55	Basophobia	0.73	0.87	Reluctant to/nervous about crossing shiny or slippery floors
5	MISC56	Basophobia	0.62	0.77	Nervous or frightened when ascending or descending some types of stairs
6	EXCITE35	Excitability	0.48	0.55	Excitable when you first arrive home, or at the dog’s kennel, after a brief absence
6	EXCITE36	Excitability	0.57	0.64	Excitable when playing with you or other familiar persons
6	EXCITE37	Excitability	0.65	0.82	Excitable just before being taken out for a walk
6	EXCITE38	Excitability	0.66	0.79	Excitable just before being taken out for work or training
7	SEPR32	Separation-related behavior	0.69	0.77	Restlessness/agitation/pacing when left alone
7	SEPR33	Separation-related behavior	0.66	0.79	Barking or whining when left alone
7	SEPR34	Separation-related behavior	0.66	0.78	Chewing/scratching at doors, floor, fencing, etc., when left alone
8	TRAIN01	Trainability	0.44	0.46	Is hard to recall when off the leash
8	TRAIN02	Trainability	0.4	0.6	Is slow to obey a ‘sit’ command
8	TRAIN03	Trainability	0.61	0.75	Is slow to obey a ‘stay’ command
8	TRAIN04	Trainability	0.6	0.48	Has difficulty attending/listening to things you say or do
9	FEAR27	Dog-directed fear	0.84	0.84	Displays fear when approached directly by an unfamiliar dog
9	FEAR31	Dog-directed fear	0.71	0.78	Displays fear when barked, growled, or lunged at by an unfamiliar dog
10	MISC52	Food focus	0.57	0.69	Begs persistently for food when people are eating
10	MISC53	Food focus	0.6	0.74	Steals food
11	FEAR26	Touch sensitivity	0.44	0.4	Displays fear when examined/treated by a veterinarian
11	FEAR29	Touch sensitivity	0.61	0.8	Displays fear when having nails trimmed, or feet touched/handled
11	FEAR30	Touch sensitivity	0.63	0.7	Displays fear when groomed or bathed
12	AGG09	Stranger-directed aggression	0.77	0.75	Barks, growls, attempts to bite when approached directly by an unfamiliar person while being walked/exercised on a leash
12	AGG10	Stranger-directed aggression	0.69	0.82	Barks, growls, attempts to bite when unfamiliar persons approach the dog when s/he is in his/her kennel
12	AGG14	Stranger-directed aggression	0.5	0.66	Barks, growls, attempts to bite when strangers walk past when the dog is in his/her home run or kennel
12	AGG15	Stranger-directed aggression	0.8	0.72	Barks, growls, attempts to bite when an unfamiliar person tries to touch or pet the dog
13	MISC60	Energy	0.61	0.72	Playful, puppyish, boisterous
13	MISC61	Energy	0.7	0.67	Active, energetic, always on the go
14	ATT39	Attachment/Attention-seeking	0.53	0.68	Displays a strong attachment for you or another familiar person
14	ATT41	Attachment/Attention-seeking	0.32	0.45	Tends to nudge or paw you (or others) for attention
15	IMP47	Impulsivity	0.59	0.63	Impulsive; does not seem to think before s/he acts
15	IMP48	Impulsivity	0.58	0.69	Becomes frustrated/impatient in a wide range of situations
15	MISC59	Impulsivity	0.59	0.41	Hyperactive, restless, has trouble settling down

Statistics describing each factor, including eigenvalue, proportion of variance (individual factor and cumulative), proportion of explained variance (individual factor and cumulative) are provided in Table 4. Eigenvalues range from 0.96 to 3.30, with eigenvalues greater than 1.0 for all factors except 14. Proportion of variance ranged from 0.2 to 0.7, and proportion of explained variance ranged from 0.03 to 0.10.

**Table 4:** Set 3 model factors with eigenvalues and proportions of explained variance.

Factor	Eigenvalues	Proportion of variance	Cumulative proportion of variance	Proportion of explained variance	Cumulative proportion of explained variance
Stranger-directed fear	3.3	0.07	0.07	0.1	0.1
Distractibility	3.21	0.06	0.13	0.1	0.2
Stranger-directed aggression	2.89	0.06	0.19	0.09	0.29
Playfulness	2.8	0.06	0.24	0.09	0.38
Basophobia	2.31	0.05	0.29	0.07	0.45
Excitability	2.27	0.05	0.34	0.07	0.52
Separation-related behavior	2.24	0.04	0.38	0.07	0.59
Dog aggression	2.17	0.04	0.42	0.07	0.66
Dog-directed fear	2.03	0.04	0.46	0.06	0.73
Trainability	1.75	0.03	0.5	0.05	0.78
Touch sensitivity	1.64	0.03	0.53	0.05	0.83
Impulsivity	1.57	0.03	0.56	0.05	0.88
Energy	1.53	0.03	0.59	0.05	0.93
Food focus	1.3	0.03	0.62	0.04	0.97
Attachment/attention-seeking	0.96	0.02	0.64	0.03	1

Two statistics were used to measure the fit of the final 15-factor model. The chi-square statistic was 4407.211 (580 DF, *p* < 0.001). The TLI was 0.76.

Table 5 shows reliabilities for each factor as measured by Cronbach’s alpha and McDonald’s omega estimates of factor saturation. Cronbach’s alpha ranged from 0.44 to 0.84. The proportion of omega due to the subscales ranged from 0.21 to 0.77. The hierarchical omega ranged from 0 to 0.57. The total omega ranged from 0.50 to 0.94.

**Table 5:** Reliabilities of factors in Set 3 (15-factor) model.

Factor	Alpha	Total omega	Hierarchical omega	Subscale omega
g	N/A	0.94	0.57	0.21
Stranger-directed fear	0.84	0.82	0.29	0.53
Playfulness	0.81	0.7	0.08	0.63
Distractibility	0.79	0.75	0.23	0.52
Dog aggression	0.75	0.82	0.13	0.69
Basophobia	0.72	0.6	0	0.6
Excitability	0.79	0.5	0.03	0.47
Separation-related behavior	0.73	0.58	0.17	0.41
Trainability	0.69	0.82	0.05	0.77
Dog-directed fear	0.8	0.84	0.37	0.47
Food focus	0.62	0.71	0.16	0.55
Touch sensitivity	0.62	0.84	0.19	0.66
Stranger-directed aggression	0.76	0.76	0.03	0.74
Energy	0.71	0.85	0.17	0.68
Attachment/attention-seeking	0.44	0.66	0.17	0.49
Impulsivity	0.68	0.83	0.22	0.61

## 4 Discussion

The C-BARQ was developed originally as a validated survey instrument for measuring behavior and temperament traits in pet dogs (30). Though designed primarily as a research tool, it has since acquired other practical uses including the diagnostic evaluation of canine behavior problems (68, 69) and the standardized behavioral assessment of various working dog populations (33–35, 70, 71). Despite its widespread use with working dogs, the basic domains of behavior measured by the C-BARQ have not changed appreciably since its original publication some 20 years ago (30). In the interim, canine scientists have identified other important dimensions of dog temperament and behavior that are likely to contribute to maintaining positive dog-human interactions, especially in the context of working partnerships. These behavioral dimensions include, but are not limited to, playfulness (36), ADHD-like traits (40–42), impulsivity and frustration intolerance (38, 39, 72), and fear associated with navigating steep or slippery walking surfaces (46, 47). The current study therefore included items aimed at measuring these additional behavioral domains to determine the factor structure and internal reliability of the resulting composite questionnaire.

A danger when adding or subtracting survey items from an existing and well-validated temperament questionnaire, such as the C-BARQ, is that these changes could disrupt the established factor structure and/or alter the interpretation of the latent personality variables extracted by exploratory factor analysis. In the current study, the subtraction of 43 of the original survey items and the introduction of 16 new items had a relatively limited impact on the original C-BARQ factor structure. Instead, most of the original 14 factors or subscales remained intact, and all the new items loaded separately onto four new subscales labeled *Playfulness*, *Impulsivity*, *Distractibility*, and *Basophobia*, respectively, though one item in the original C-BARQ’s *Trainability* factor—“dog is easily distracted by interesting sights, sounds, smells”—loaded more strongly on the new *Distractibility* factor, and one item in *Nonsocial Fear*—“Fearful when first exposed to unfamiliar situations (e.g., novel environments, first visit to the veterinarian, etc.)”—loaded on the *Stranger-directed Fear* factor. Additionally, two items from the ‘Miscellaneous’ section of the C-BARQ addressing food stealing and food begging behavior formed another new factor we labeled *Food Focus*. Most of these factors, apart from some of the 2- or 3-item factors (*Attachment/Attention-seeking*, *Food Focus*, and *Touch Sensitivity*) obtained good-adequate internal reliabilities (see Table 5).

Two of the original C-BARQ factors, *Owner-directed Aggression* and *Nonsocial Fear*, failed to factor out in this detection dog sample, and the items representing the original C-BARQ factors, *Dog-directed Aggression* and *Dog Rivalry*, loaded onto a single factor labeled *Dog Aggression* in the new questionnaire. While it is possible that the reduction in the total number of aggression and fear items in the WDC-BARQ contributed to the loss of these original factors, the continued reliability of the *Stranger-directed Fear* and *Stranger-directed Aggression* factors (Table 5) would tend to argue against this. The most likely explanation for the loss of *Owner-directed Aggression* as a coherent factor in the WDC-BARQ is the low number of scores greater than zero (88% zero values) for all the items loading on this factor (see Supplementary Table 3). Since aggression toward owners/handlers would interfere substantially with the performance of working detection dogs, it is likely that the sample of dogs assayed in the present study was pre-selected to show extremely low levels of this behavior. Nonetheless, given the importance of this behavior in the selection and deployment of working dogs, it probably makes sense to retain these aggression items in the WDC-CBARQ for research and dog selection purposes.

The communality estimates and factor loadings for the *Nonsocial Fear* items were too low to be factorable in this sample of dogs (Supplementary Table 3), and one of the original items “Fearful when first exposed to unfamiliar situations (e.g., novel environments, first visit to the veterinarian, etc.”) loaded more strongly on the *Stranger-directed Fear* factor. Despite this discrepancy, the coherence and reliability of this factor in other dog populations (30, 33), and the importance of being able to detect and measure environmental fears in potential working dogs, would argue for retaining and possibly supplementing these items in the WDC-BARQ. Again, it should be emphasized that the dogs included in the current study were either in training, actively working, or retired, and therefore had already been selected for suitability for detection work. Dogs that demonstrated appreciable levels of nonsocial fear or owner-directed aggression would likely have been eliminated before starting their careers, and traits that lack variation within a sample cannot contribute to factor analysis. However, future studies should seek to validate the inclusion of these types of aggression and fear factors in the WDC-BARQ by exploring the factor structure of the questionnaire among younger dogs prior to their selection for working roles.

Similarly, while the three original *Chasing* items were inadvertently omitted from the first phase of data collection and were therefore excluded from the factor analysis, the consistently high internal reliability of the *Chasing* subscale in other C-BARQ studies (10, 30) and the relevance of this behavior to working performance, suggests that these items should also be included in the WDC-BARQ. Hopefully, future studies will be able to confirm its validity as a distinct factor in detection dogs.

The choice of how many factors to include in the final model was based on a number of considerations. While MAP and Parallel Analysis generally recommended 11 or 12 factors, acceptance of these or the 13-factor model would have involved the loss or disintegration of several original C-BARQ factors (e.g., *Touch Sensitivity, Attachment/Attention-seeking, and Energy*) in addition to *Owner-directed aggression and Nonsocial fear*. The new *Impulsivity* factor also failed to factor out in the 13-factor model. The 14-factor model excluded *Attachment/Attention-seeking*, and one of the *Touch Sensitivity items* (“fear of veterinary examinations”) loaded on the *Stranger-directed Fear* factor. The 15- and 16-factor models were nearly identical and included all of the original C-BARQ factors (except *Owner-directed Aggression and Nonsocial Fear*) as well as the five new factors. However, since one of the primary goals of factor analysis is data reduction, the 15-factor model was deemed to be the most appropriate and informative for future use. Because it includes most of the original C-BARQ factors, the adoption of this model for canine behavioral assessments should also facilitate comparisons with the findings of previous studies that have used the C-BARQ to evaluate behavior in dogs.

The new factors extracted in the WDC-BARQ analysis generally concur with the findings of previous studies. For example, the motivation to engage in play behavior (ball/toy retrieval, tug-of-war games, etc.) has been used for decades to evaluate and predict working dog performance (20, 24, 28, 73). Svartberg (36) identified two distinct “playfulness” factors in a sample of Swedish dogs, one of which was human-directed and the other dog-directed. The former was associated with survey items relating to a dog’s tendency to play with and retrieve objects/toys and its eagerness to play with familiar people, and resembles the WDC-BARQ’s *Playfulness* factor, except that the latter addresses the dog’s eagerness to play with new or unfamiliar people rather than familiar ones. In the future, it will be interesting to determine if dogs’ scores on this factor reliably predict success in training for detection work.

Although poor impulse control and inattention/distractibility are both commonly regarded as symptoms of ADHD in both dogs and humans (40–42), the impulsivity and distractibility items in the WDC-BARQ factored-out separately in the current analysis, suggesting that they may be measuring different behavioral domains. The 3-item *Impulsivity* factor comprises items that focus on hyperactivity in combination with impulsiveness and low frustration tolerance and may describe the canine equivalent of a ‘Type A’ dog who is intensely focused and goal-directed, perseverative, and frustrated by obstacles (e.g., cannot sit still, rushes through doorways, demand barks, grabs at toys, etc.). In some respects, this factor may be functionally equivalent to the concept of “drive” which is generally perceived as a desirable trait, at least in detection dogs, although excessive drive could also be undesirable when associated with a lack of inhibitory control (38). In contrast, the 6-item *Distractibility* factor seems to describe the opposite: i.e., a dog that is so easily distracted by environmental stimuli (people, other dogs, sounds, odors, and so on) that it is unable to maintain a sustained focus on anything. Looking forwards, it would be valuable to determine if these putative personality types can be validated experimentally using appropriate tests, such as the cylinder test (38, 72) or the impossible task (74–76), and whether scores on these traits can enhance prediction of certification and/or performance outcomes in different types of working dogs.

Fears of stairs, sidewalk grates, and/or slippery floors are widely recognized as significant behavioral issues in working dogs (20, 46, 47) but relatively little research has been directed toward understanding the etiology or ontogeny of these fears or how they may relate to one another. In the working dog community,^2^ fear of stairs is generally viewed as distinct from fear of grates or slippery floors (sometimes referred to collectively as “underfootings”), but the current analysis suggests they may be related, possibly by a common fear of falling in these different contexts. For this reason, the label “Basophobia” (defined as ‘fear of falling’) has been applied to this factor, although further research will be needed to confirm the validity of this proposed new terminology. It should also be noted that the items in this factor did not associate with any of the C-BARQ *Nonsocial Fear* items (e.g., fear of loud noises, traffic, unfamiliar objects, etc.) suggesting a different causation, perhaps involving balance or proprioception issues (20).

It is unclear how useful the new *Food focus* factor will be in the behavioral assessment of detection and other working dogs, since many working dog groups tend to eschew the use of food rewards as primary reinforcers in training in operational settings. *Food focus* could, however, be an important parameter in learning an odor, as food is frequently used in the initial training of an odor (77). Furthermore, identifying and utilizing the most motivating reward for each dog, whether food or toy, could help to enlarge the available pool of potential detection dogs (78). In other working contexts, such as guiding or assistance work, a strong focus on food items might also be associated with undesirable scavenging behavior.

As with any measure of canine behavior, the proposed WDC-BARQ has its limitations. First, the assessor must have sufficient knowledge of the dog to complete the questionnaire, so the instrument is unlikely to aid in the initial selection and purchase of dogs from external suppliers. Because it is a proxy assessment, its reliability and validity ultimately depend on the assessors’ experience and accuracy at ‘reading’ dog behavior and their ability or willingness to provide objective and unbiased responses to the various survey items. Further research will therefore be needed to determine the convergent and predictive validity of the new instrument’s subscales (factors) as well as their inter-rater and test–retest reliability.

The presence of a number of 2- and 3-item factors, as well as some factors with low reliability is also undesirable. Factor analysis researchers recommend that a minimum of four items is necessary to determine each factor (63). Consideration should be given to enhancing their internal reliability by adding further items to these subscales. We calculated both alpha and omega as measures of the reliability of the factors in this study. Although frequently reported in factor analysis studies, alpha’s use has been criticized because it does not reflect the internal structure of the test. This value can underestimate the reliability of the scale and overestimate the fraction of the test variance associated with the general variance in the test. Model-based estimates such as common variance shared by all items (total omega), the amount of variance associated with the factors, and unique variance (and error) for individual items are preferred because all the data in the survey are used.

Alpha values for the final 15-factor model ranged from 0.44 to 0.84 (Table 5). The factors with alpha values less than 0.7, indicating low reliability (85) are *Trainability* (0.69), *Food focus* (0.62), *Touch sensitivity* (0.62), *Attachment/Attention-seeking* (0.44) and *Impulsivity* (0.68). *Food focus* and *Attachment/Attention-seeking* contained two items, while *Touch sensitivity* and *Impulsivity* contained three items. Since reliability is related to the number of items in a factor, future work should identify additional items that can be used to measure these behaviors. The low reliability of *Trainability* may warrant further research to determine whether it is composed of multiple real behaviors. It is challenging to define in a population pre-selected for odor detection, in part because of removed *Trainability* items that were invariant in the studied population. These included TRAIN05 (Is slow to respond to corrections or reprimands: thick-skinned), TRAIN06 (Is slow to learn new tricks or tasks), and TRAIN08 (Is uninterested in ‘fetching’ or attempting to fetch sticks, toys, balls, or objects). Improvements could be made by adding new items related to trainability.

The subscale omega value for the shared variance among all items is 0.21. The omega values for the 15 subscales/factors range from 0.41 to 0.77. These quantities are not comparable with alpha and are expected to be lower because the common variance associated with all the items is not included in the subscale omega. While there are few guidelines for interpreting subscale omega, some authors have suggested that 0.50 is minimally acceptable and 0.75 is preferable. In the present study, low omega subscale values are associated with *Stranger-directed fear*, *Excitability*, *Separation-related behavior*, *Dog-directed fear*, and *Attachment/attention-seeking*. Some of the low values may be related to the small numbers of items. Future research could address adding items to these factors to strengthen them. It is unclear why *Stranger-directed fear* and *Excitability*, which both have four items, have low reliability and whether this is related to the pre-selection of odor detection dogs for this study. With the exception of *Attachment/attention-seeking*, the set of factors with low reliabilities is different for alpha and omega. This is consistent with the different computational methods and interpretations of the two statistics. These behaviors may vary less in pre-selected odor detection dogs than in the general dog population.

We were unable to account for the influence of demographic factors in our analyses because of the relatively large sample size needed when analyzing ordinal data sets (66). Data characteristics that impact the required sample size include item communalities and number of items per factor (55). In the present study, the communalities are mostly moderate in magnitude and the number of items per factor is sometimes small. Including additional variables such as breed or breed group would result in subgroups too small to be meaningfully analyzed.

The factor structure of the data analyzed is partially dependent on the population of dogs surveyed. Most of the dogs in the current survey worked in human scent detection, while there were fewer explosives detection dogs (approximately 10%) (Table 2). Differences in working environment among these dogs may have influenced the types of behaviors observed. For example, USAR dogs work in areas cluttered with debris and rubble, while explosives detection dogs often operate in transportation centers containing noisy crowds of people, equipment, and vehicles. As a result, behaviors with low variation in a population of dogs with human detection roles may be more prevalent in explosives detection dogs, and vice versa. When the full range of working dog types is considered, human selection for performance in different specialized roles and environments would likely be a further source of behavioral variation. Given this variation in dog types and functions, future studies should aim to validate the WDC-BARQ in a range of different working dog populations.

Despite these limitations, and the fact that it was developed using a sample of working detection dogs, we believe the new subscales of the WDC-BARQ are potentially of universal relevance to the behavioral and temperamental assessment of most working dogs.

## Data availability statement

The original contributions presented in the study are included in the article/Supplementary material. For access to the raw data please contact the corresponding author.

## Ethics statement

The studies involving humans were approved by University of Pennsylvania Institutional Review Board (IRB). The studies were conducted in accordance with the local legislation and institutional requirements. The Ethics Committee/Institutional Review Board waived the requirement of written informed consent for participation from the participants or the participants’ legal guardians/next of kin because no personal information regarding human participants was collected or retained.

## Author contributions

EH: Conceptualization, Formal analysis, Methodology, Validation, Writing – original draft, Writing – review & editing, Data curation, Investigation. JE: Conceptualization, Project administration, Writing – original draft, Writing – review & editing, Investigation. CO: Conceptualization, Supervision, Writing – review & editing, Funding acquisition, Investigation, Project administration. DE: Data curation, Project administration, Writing – review & editing. JS: Conceptualization, Formal analysis, Funding acquisition, Investigation, Methodology, Project administration, Supervision, Validation, Writing – original draft, Writing – review & editing.

## Footnotes

^1^ aafs.org

^2^ https://www.iwdr.org/master-knowledge-base/4-fear-of-underfootings/
