# Supplementary material for: Development of a modified C-BARQ for evaluating behavior in working dogs
Source: Front Vet Sci. 2024 Jun 28;11:1371630. doi: 10.3389/fvets.2024.1371630 (PMC11239546; doi:10.3389/fvets.2024.1371630)
Supplement: Supplementary file 3 [file Table_1.docx]

Supplementary Material

Development of a Modified C-BARQ for Evaluating Behavior in Working Dogs

**Authors:** Hare, Elizabeth^1,2^, Essler, J.^3^, Otto, C.M.,^1^ Ebbecke, D,^1^ Serpell, J.A.^1*^*****

**Correspondence:** Corresponding Author: [serpell@upenn.edu](mailto:serpell@upenn.edu)

# Supplementary Data

Supplementary Data Table 1. Polychoric correlations for raw questionnaire data. Correlations were computed for each pair of ordinal responses in the questionnaire (see spreadsheet).

Supplementary Data Table 2. Polychoric correlations for questionnaire data after imputation of missing values (see spreadsheet).

# Supplementary Tables

Supplementary Table 1. List of original C-BARQ and new or modified WDC-BARQ items.

**Original C-BARQ Working Dog C-BARQ (prototype)**

| C-BARQ factor: **Trainability** |  |
| --- | --- |
| - Dog returns immediately when called while off leash | - Dog is hard to recall when off the leash (TRAIN01) |
| - Dog obeys a “sit” command immediately | - Dog is slow to obey a “sit” command (TRAIN02) |
| - Dog obeys a “stay” command immediately | - Dog is slow to obey a “stay” command (TRAIN03) |
| - Dog seems to attend to or listen closely to everything the owner says or does | - Dog has difficulty attending/listening to things you say or do (TRAIN04) |
| - Dog is slow to respond to correction or reprimands | - Dog is slow to respond to correction or reprimands (TRAIN05) |
| - Dog is slow to learn new tricks or tasks | - Dog is slow to learn new tricks or tasks (TRAIN06) |
| - Dog is easily distracted by interesting sights, sounds, or smells | - Unfocused; dog is easily distracted by interesting sights, sounds or smells (TRAIN07) |
| - Dog will fetch or attempt to fetch sticks, balls, and other objects | - Dog is uninterested in “fetching” or attempting to fetch sticks, toys, balls, or objects (TRAIN08). |

| C-BARQ factor: **Stranger-directed aggression** |  |
| --- | --- |
| *Dog acts aggressively (barks, growls, bites or attempts to bite)* |  |
| - When approached directly by an unfamiliar adult while being walked or exercised on a leash. | - When approached directly by an unfamiliar person while being walked or exercised on a leash (AGG09). |
| - When approached directly by an unfamiliar child while being walked or exercised on a leash |  |
| - Toward unfamiliar persons approaching the dog while it is in the owner’s car | - When unfamiliar persons approach the dog when s/he is (a) in your car (e.g., at the gas station) or (b) in his/her kennel (AGG10) |
| - When an unfamiliar person approaches the owner or a member of the owner’s family at home |  |
| - When an unfamiliar person approaches the owner or a member of the owner’s family away from home |  |
| - When mailmen or other delivery workers approach the home |  |
| - When strangers walk past the home while the dog is in the yard | - When strangers walk past when the dog is in his/her home run or kennel (AGG14) |
| - When an unfamiliar person tries to touch or pet the dog | - When an unfamiliar person tries to touch or pet the dog (AGG15) |
| - When joggers, cyclists, roller skaters, or skateboarders pass the home while the dog is in the yard |  |
| - Toward unfamiliar persons visiting the home |  |

| C-BARQ factor: **Owner-directed aggression** |  |
| --- | --- |
| *Dog acts aggressively (barks, growls, bites or attempts to bite)* |  |
| - When verbally corrected or reprimanded by a member of the household |  |
| - When toys, bones, or other objects are taken away by a member of the household | - When toys, bones or other objects are taken away by you or another familiar person (AGG11) |
| - When bathed or groomed by a member of the household |  |
| - When approached directly by a member of the household while it is eating | - When you or another familiar person approaches the dog directly while s/he is eating (AGG12) |
| - When food is taken away by a member of the household | - When his/her food is taken away by you or another familiar person (AGG13) |
| - When stared at directly by a member of the household | - When stared at directly by you or another familiar person (AGG17) |
| - When stepped over by a member of the household |  |
| - When a member of the household retrieves food or objects stolen by the dog |  |

| C-BARQ factor: **Dog rivalry** |  |
| --- | --- |
| *Dog acts aggressively (barks, growls, bites or attempts to bite)* |  |
| - Towards another (familiar) dog in your household. | - Towards other familiar dogs (AGG19) |
| - When approached at a favorite resting/sleeping place by another household dog. |  |
| - When approached while eating by another household dog. |  |
| - When approached while playing with/chewing a favorite toy, bone, object by another household dog. | - When approached while playing with/chewing a favorite toy, bone, object, etc., by another familiar dog (AGG20) |

| C-BARQ factor: **Dog-directed aggression** |  |
| --- | --- |
| *Dog acts aggressively (barks, growls, bites or attempts to bite)* |  |
| - When approached directly by an unfamiliar male dog while being walked or exercised on a leash | - When approached directly by an unfamiliar dog while being walked/exercised on a leash (AGG16) |
| - When approached directly by an unfamiliar female dog while being walked or exercised on a leash |  |
| - Toward unfamiliar dogs visiting the home |  |
| - When barked, growled, or lunged at by an unfamiliar dog. | - When barked, growled, or lunged at by another dog (AGG18). |

| C-BARQ factor: **Stranger-directed fear** |  |
| --- | --- |
| *Dog acts anxious or fearful* |  |
| - When approached directly by an unfamiliar adult while away from the home | - When an unfamiliar person approaches the dog when s/he is away from his/her normal home environment or kennel (FEAR21) |
| - When approached directly by an unfamiliar child while away from the home |  |
| - When unfamiliar persons visit the home | - When an unfamiliar person visits your home or approaches the dog when in his/her home kennel (FEAR23) |
| - When an unfamiliar person tries to touch or pet the dog | - When an unfamiliar person tries to touch or pet the dog (FEAR24) |

| C-BARQ factor: **Dog-directed fear** |  |
| --- | --- |
| *Dog acts anxious or fearful* |  |
| - When approached directly by an unfamiliar dog of the same or larger size | - When approached directly by an unfamiliar dog (FEAR27) |
| - When approached directly by an unfamiliar dog of a smaller size |  |
| - When unfamiliar dogs visit the home. |  |
| - When barked, growled, or lunged at by an unfamiliar dog | - When barked, growled, or lunged at by an unfamiliar dog (FEAR31) |

| C-BARQ factor: **Nonsocial fear** |  |
| --- | --- |
| *Dog acts anxious or fearful……* |  |
| - In response to sudden or loud noises (e.g., vacuum cleaner, car backfire, road drills, objects being dropped, etc.). | - In response to sudden or loud noises (e.g., gun fire, car backfire, road drills, objects being dropped, etc.) (FEAR22). |
| - In heavy traffic |  |
| - In response to strange or unfamiliar objects on or near the sidewalk (e.g., plastic trash bags, leaves, litter, flags flapping, etc.). | - In response to strange or unfamiliar objects on or near sidewalks or walkways (e.g., plastic trash bags, leaves, litter, flags flapping, etc.) (FEAR25). |
| - During thunderstorms, firework displays, or similar |  |
| - When first exposed to unfamiliar situations (e.g., first car trip, first visit to the veterinarian, etc.). | - When first exposed to unfamiliar situations (e.g., novel environments, first visit to the veterinarian, etc.) (FEAR28). |
| - In response to wind or wind-blown objects |  |

| C-BARQ factor: **Touch sensitivity** |  |
| --- | --- |
| *Dog acts anxious or fearful* |  |
| - When examined or treated by a veterinarian | - When examined or treated by a veterinarian (FEAR26) |
| - When having its nails clipped by a household member | - When having nails trimmed, or feet touched/handled (FEAR29) |
| - When groomed or bathed by a household member. | - When groomed or bathed (FEAR30) |
| - When having feet toweled by a household member |  |

| C-BARQ factor: **Separation-related behavior** |  |
| --- | --- |
| *Dog displays* |  |
| - Shaking, shivering or trembling when left or about to be left on its own |  |
| - Excessive salivation when left or about to be left on its own |  |
| - Restlessness/agitation/pacing when left or about to be left on its own | - Restlessness/agitation/pacing when left alone (SEP32) |
| - Whining when left or about to be left on its own | - Barking or whining when left alone (SEP33) |
| - Barking when left or about to be left on its own |  |
| - Howling when left or about to be left on its own |  |
| - Chewing or scratching at doors, floor, windows, and curtains when left or about to be left on its own | - Chewing/scratching at doors, floor, fencing, etc., when left alone (SEP34) |
| - Loss of appetite when left or about to be left on its own |  |

| C-BARQ factor: **Excitability** |  |
| --- | --- |
| *Dog over-reacts or is excitable* |  |
| - When a member of the household returns home after a brief absence | - When you first arrive home, or at the dog’s kennel, after a brief absence (EXCITE35) |
| - When playing with a member of the household | - When playing with you or other familiar persons (EXCITE36 |
| - When the doorbell rings |  |
| - Just before being taken for a walk | - Just before being taken out for a walk (EXCITE37 |
| - Just before being taken on a car trip | - Just before being taken out for work or training (EXCITE38) |
| - When visitors arrive at its home |  |

| C-BARQ factor: **Attachment/Attention-seeking** |  |
| --- | --- |
| - Dog displays a strong attachment for a particular member of the household | - Dog displays a strong attachment for you or another familiar person (ATT39) |
| - Dog tends to follow a member of household from room to room about the house | - Dog is unwilling to leave your side, even when working; reluctant to work at a distance from you/the handler (ATT40 |
| - Dog tends to sit close to or in contact with a member of the household when that individual is sitting down |  |
| - Dog tends to nudge, nuzzle, or paw a member of the household for attention when that individual is sitting down | - Dog tends to nudge or paw you (or others) for attention (ATT41) |
| - Dog becomes agitated (whines, jumps up, tries to intervene) when a member of the household shows affection for another person | - Dog becomes agitated (whines, jumps up, tries to intervene) when attention is given to another person or animal (ATT42 |
| - Dog becomes agitated (whines, jumps up, tries to intervene) when a member of the household shows affection for another dog or animal |  |

| C-BARQ factor: **Chasing** |  |
| --- | --- |
| - Dog acts aggressively toward cats, squirrels, and other animals entering its yard |  |
| - Dog chases or would chase cats given the opportunity | - Dog chases or would chase cats given the opportunity (Not included) |
| - Dog chases or would chase birds given the opportunity | - Dog chases or would chase birds given the opportunity (Not included) |
| - Dog chases or would chase squirrels, rabbits and other small animals given the opportunity | - Dog chases or would chase squirrels, rabbits and other small animals given the opportunity (Not included) |

| C-BARQ factor: **Energy** |  |
| --- | --- |
| - Dog is playful, puppyish, boisterous | - Dog is playful, puppyish, boisterous (MISC60) |
| - Dog is active, energetic, always on the go | - Dog is active, energetic, always on the go (MISC61) |

| **Miscellaneous Items**—excluding Chasing and Energy items (see above) |  |
| --- | --- |
| - Dog escapes or would escape from home or yard given the chance | - Dog escapes or would escape from home, yard or kennel given the chance (MISC51) |
| - Dog rolls in animal droppings or other ‘smelly’ substances |  |
| - Dog eats own or other animals’ droppings or feces |  |
| - Dog chews inappropriate objects |  |
| - Dog ‘mounts’ objects, furniture, or people |  |
| - Dog begs persistently for food when people are eating | - Dog begs persistently for food when people are eating (MISC52) |
| - Dog steals food | - Dog steals food (MISC53) |
|  | - Dog is reluctant to, or nervous about, crossing grates or other unfamiliar surfaces* (MISC54) |
|  | - Dog is reluctant to, or nervous about, crossing shiny or slippery floors* (MISC55) |
| - Dog is nervous or frightened on stairs | - Dog is nervous or frightened when ascending or descending some types of stairs (MISC56) |
| - Dog pulls excessively hard when on leash | - Dog pulls excessively hard when on leash (MISC57) |
| - Dog urinates against objects/ furnishings indoors | - Dog urinates against objects/ furnishings indoors (MISC58) |
| - Dog urinates when approached, petted, handled, or picked up. |  |
| - Dog urinates when left alone at night, or during the daytime |  |
| - Dog defecates when left alone at night, or during the daytime |  |
| - Dog is hyperactive, restless, has trouble settling down | - Dog is hyperactive, restless, has trouble settling down (MISC59) |
| - Dog stares intently at nothing visible |  |
| - Dog snaps at (invisible) flies |  |
| - Dog chases own tail/hind end | - Dog chases own tail/hind end (MISC68) |
| - Dog chases/follows shadows, light spots, etc. | - Dog chases/follows shadows, light spots, etc. (MISC69) |
| - Dog barks persistently when alarmed or excited | - Dog barks persistently when alarmed or excited (MISC70) |
| - Dog licks him/herself excessively |  |
| - Dog licks people or objects excessively | - Dog licks people or objects excessively (MISC71) |
| - Dog displays other bizarre, strange, or repetitive behavior(s) |  |

| New items: **Playfulness** |  |
| --- | --- |
|  | - Dog eagerly engages in play with new/unfamiliar people*(PLAY43) |
|  | - Dog is highly toy-focused; attention riveted on tug-toys/balls when these are held by handler or other persons* (PLAY44) |
|  | - Dog eagerly initiates play sessions; brings objects/toys to you/the handler and retrieves them when thrown* (PLAY45) |
|  | - Dog hunts persistently for thrown or hidden toys/objects; not easily distracted from this task* (PLAY46) |

| New items: **Impulsivity** |  |
| --- | --- |
|  | - Dog is impulsive; doesn’t seem to think before s/he acts* (IMP47) |
|  | - Dog becomes frustrated/impatient in a wide range of situations* (IMP48) |
|  | - Dog is difficult to interrupt or distract when doing things s/he wants to do* (IMP49 |
|  | - Dog displays repetitive behavior (circling, pacing, barking, tail-chasing, etc.) when unable to access something s/he wants* (IMP50) |

| New items: **Distractibility** |  |
| --- | --- |
|  | - Dog becomes highly excited and/or distracted when encountering unfamiliar dogs* (MISC62) |
|  | - Dog becomes highly excited and/or distracted when encountering unfamiliar people* (MISC63) |
|  | - When working, dog is easily distracted or preoccupied by odors—engages in persistent sniffing of ground or objects* (MISC64) |
|  | - Dog has difficulty shifting attention away from interesting or distracting stimuli (e.g., other dogs, odors, people, small animals, etc.)* (MISC65) |
|  | - Dog is distracted or nervous in new, unfamiliar environments; has difficulty maintaining focus on work* (MISC66) |
|  | - Dog is slow to recover after being distracted, startled, or frightened—takes a long time to resume normal activity or work* (MISC67) |

|  |  |  |  |  |  |  |  |
| --- | --- | --- | --- | --- | --- | --- | --- |

Supplementary Table 2. Recommended number of factors by PA and MAP

|  |  |  | |
| --- | --- | --- | --- |
| **Analysis Set** | Data Set | Parallel Analysis | Minimum Average Partial |
| **1** | Complete imputed | 16 | 13 |
| **2** | drop12 | 11 | 9 |
| **2** | drop13 | 11 | 11 |
| **2** | drop14 | 11 | 9 |
| **2** | drop15 | 12 | 11 |
| **2** | drop16 | 12 | 11 |
| **2** | drop17 | 13 | 11 |
| **2** | drop18 | 13 | 11 |
| **3** | drop15 |  |  |

Supplementary Table 3. Survey items removed after first set of factor analyses with reasons for removal.

| **Item** | **12-Factor Model** | **13-Factor Model** | **14-Factor Model** | **15-Factor Model** | **16-Factor Model** | **17-Factor Model** | **18-Factor Model** |
| --- | --- | --- | --- | --- | --- | --- | --- |
| **TRAIN02** | communality < 0.40 | communality < 0.40 | communality < 0.40 |  |  |  |  |
| **TRAIN05** | communality < 0.40 | communality < 0.40 | communality < 0.40 | communality < 0.40 | communality < 0.40 | communality < 0.40 | communality < 0.40 |
| **TRAIN06** | communality < 0.40 | communality < 0.40 | communality < 0.40 | communality < 0.40 | communality < 0.40 | communality < 0.40 | communality < 0.40 |
| **TRAIN08** | highest loading < 0.32 | highest loading < 0.32 | highest loading < 0.32 | highest loading < 0.32 | highest loading < 0.32 | highest loading < 0.32 | highest loading < 0.32 |
| **AGG11** | Frequency of 0 values > 88% | Frequency of 0 values > 88% | Frequency of 0 values > 88% | Frequency of 0 values > 88% | Frequency of 0 values > 88% | Frequency of 0 values > 88% | Frequency of 0 values > 88% |
| **AGG12** | Frequency of 0 values > 88% | Frequency of 0 values > 88% | Frequency of 0 values > 88% | Frequency of 0 values > 88% | Frequency of 0 values > 88% | Frequency of 0 values > 88% | Frequency of 0 values > 88% |
| **AGG13** | Frequency of 0 values > 88% | Frequency of 0 values > 88% | Frequency of 0 values > 88% | Frequency of 0 values > 88% | Frequency of 0 values > 88% | Frequency of 0 values > 88% | Frequency of 0 values > 88% |
| **AGG17** | Frequency of 0 values > 88% | Frequency of 0 values > 88% | Frequency of 0 values > 88% | Frequency of 0 values > 88% | Frequency of 0 values > 88% | Frequency of 0 values > 88% | Frequency of 0 values > 88% |
| **FEAR22** | communality < 0.40, highest loading < 0.32 | communality < 0.40 | communality < 0.40, highest loading < 0.32 | communality < 0.40, highest loading < 0.32 |  |  |  |
| **FEAR25** |  |  |  | highest loading < 0.32 |  |  |  |
| **FEAR29** | communality < 0.40 |  |  |  |  |  |  |
| **ATT39** | communality < 0.40,highest loading < 0.32 | communality < 0.40, highest loading < 0.32 | communality < 0.40, highest loading < 0.32 |  |  |  |  |
| **ATT40** | communality < 0.40 | communality < 0.40 | communality < 0.40 | communality < 0.40 | communality < 0.40 |  |  |
| **ATT41** | communality < 0.40 | communality < 0.40 | communality < 0.40 |  |  |  |  |
| **ATT42** | communality < 0.40 | communality < 0.40 | communality < 0.40 | communality < 0.40 | communality < 0.40 | communality < 0.40, highest loading < 0.32 | communality < 0.40, highest loading < 0.32 |
| **IMP49** | communality < 0.40 | communality < 0.40 | communality < 0.40 | communality < 0.40 | communality < 0.40 | communality < 0.40 |  |
| **MISC50** | communality < 0.40, highest loading < 0.32 | communality < 0.40, highest loading < 0.32 | communality < 0.40, highest loading < 0.32 | highest loading < 0.32 | communality < 0.40, highest loading < 0.32 | communality < 0.40, highest loading < 0.32 | communality < 0.40 |
| **MISC51** | communality < 0.40, highest loading < 0.32 | communality < 0.40,highest loading < 0.32 | communality < 0.40 ,highest loading < 0.32 | highest loading < 0.32 |  |  | highest loading < 0.32 |
| **MISC57** | communality < 0.40, highest loading < 0.32 | communality < 0.40, highest loading < 0.32 | «, highest loading < 0.32 | communality < 0.40, highest loading < 0.32 | communality < 0.40,highest loading < 0.32 | communality < 0.40, highest loading < 0.32 | communality < 0.40, highest loading < 0.32 |
| **MISC58** | communality < 0.40 | communality < 0.40 | communality < 0.40 | communality < 0.40, highest loading < 0.32 | communality < 0.40 | communality < 0.40, highest loading < 0.32 | communality < 0.40 |
| **MISC60** | communality < 0.40 | communality < 0.40 |  |  |  |  |  |
| **MISC63** | communality < 0.40 | communality < 0.40 | communality < 0.40 | communality < 0.40 | communality < 0.40 |  |  |
| MISC68 | communality < 0.40, highest loading < 0.32 |  |  |  |  |  | communality < 0.40 |
| **MISC69** | communality < 0.40 | communality < 0.40 | communality < 0.40 |  |  |  |  |
| **MISC70** | communality < 0.40 | communality < 0.40 | communality < 0.40, highest loading < 0.32 | communality < 0.40 | communality < 0.40 | communality < 0.40 | communality < 0.40 |
| **MISC71** | highest loading < 0.32 | communality < 0.40, highest loading < 0.32 | communality < 0.40, highest loading < 0.32 | communality < 0.40, highest loading < 0.32 | communality < 0.40, highest loading < 0.32 | communality < 0.40, highest loading < 0.32 | communality < 0.40, highest loading < 0.32 |

Supplementary Table 4. Breed distribution of participating dogs

| **Breed** | **N (%)** |
| --- | --- |
| Breed |  |
| Airedale Terrier | 1 (<0.1%) |
| Akita | 1 (<0.1%) |
| Alaskan Malamute | 1 (<0.1%) |
| American Coonhound | 2 (0.2%) |
| American Indian Dog | 1 (<0.1%) |
| American Pit Bull Terrier | 5 (0.4%) |
| American Water Spaniel | 1 (<0.1%) |
| Anatolian Shepherd | 2 (0.2%) |
| Australian Cattle Dog | 8 (0.7%) |
| Australian Kelpie | 7 (0.6%) |
| Australian Koolie | 2 (0.2%) |
| Australian Shepherd | 17 (1.5%) |
| Australian Shepherd (Miniature) | 2 (0.2%) |
| Beagle | 2 (0.2%) |
| Beauceron | 1 (<0.1%) |
| Belgian Groenendael | 1 (<0.1%) |
| Belgian Malinois | 123 (11%) |
| Belgian Malinois x Dutch Shepherd cross | 1 (<0.1%) |
| Belgian Sheepdog | 1 (<0.1%) |
| Belgian Tervuren | 6 (0.5%) |
| Berger Blanc Suisse (Swiss Shepherd Dog) | 1 (<0.1%) |
| Bernese Mountain Dog | 2 (0.2%) |
| Black and Tan Coonhound | 1 (<0.1%) |
| Bloodhound | 8 (0.7%) |
| Blue Tick Coonhound | 1 (<0.1%) |
| Border Collie | 45 (4.0%) |
| Border Terrier | 2 (0.2%) |
| Bouvier des Flandres | 2 (0.2%) |
| Boxer | 3 (0.3%) |
| Boykin Spaniel | 1 (<0.1%) |
| Briard | 1 (<0.1%) |
| Brittany | 4 (0.4%) |
| Bull Terrier | 1 (<0.1%) |
| Cardigan Welsh Corgi | 1 (<0.1%) |
| Carolina Dog | 1 (<0.1%) |
| Catahoula Leopard Dog | 3 (0.3%) |
| Cavalier King Charles Spaniel | 1 (<0.1%) |
| Chesapeake Bay Retriever | 3 (0.3%) |
| Clumber Spaniel | 1 (<0.1%) |
| Cocker Spaniel (American) | 2 (0.2%) |
| Cocker Spaniel (English) | 8 (0.7%) |
| Collie | 1 (<0.1%) |
| Collie (Rough) | 1 (<0.1%) |
| Cross-breed | 92 (8.2%) |
| Curly-Coated Retriever | 1 (<0.1%) |
| Dachshund | 2 (0.2%) |
| Dachshund (Miniature) | 1 (<0.1%) |
| Dalmatian | 2 (0.2%) |
| Doberman Pinscher | 10 (0.9%) |
| Dutch Shepherd | 53 (4.7%) |
| English Coonhound | 1 (<0.1%) |
| English Shepherd | 2 (0.2%) |
| English Springer Spaniel | 14 (1.3%) |
| Eurasier | 1 (<0.1%) |
| Field Spaniel | 1 (<0.1%) |
| Flat-Coated Retriever | 4 (0.4%) |
| Fox Terrier (Smooth) | 2 (0.2%) |
| German Pinscher | 1 (<0.1%) |
| German Shepherd | 171 (15%) |
| German Shorthaired Pointer | 85 (7.6%) |
| German Wirehaired Pointer | 3 (0.3%) |
| Golden Retriever | 29 (2.6%) |
| Gordon Setter | 2 (0.2%) |
| Hovawart | 1 (<0.1%) |
| Ibizan Hound | 1 (<0.1%) |
| Icelandic Sheepdog | 1 (<0.1%) |
| Irish Setter | 1 (<0.1%) |
| Jack Russell Terrier | 1 (<0.1%) |
| Jagd Terrier | 1 (<0.1%) |
| Keeshond | 1 (<0.1%) |
| Kooikerhondje | 3 (0.3%) |
| Labrador Retriever | 214 (19%) |
| Lagotto Romagnolo | 1 (<0.1%) |
| Lakeland Terrier | 1 (<0.1%) |
| Lancashire Heeler | 1 (<0.1%) |
| Leonberger | 1 (<0.1%) |
| McNab Shepherd | 4 (0.4%) |
| Miniature American Shepherd | 1 (<0.1%) |
| Mixed-breed | 70 (6.3%) |
| Newfoundland | 1 (<0.1%) |
| Nova Scotia Duck Tolling Retriever | 4 (0.4%) |
| Papillon | 1 (<0.1%) |
| Parson Russell Terrier | 1 (<0.1%) |
| Pembroke Welsh Corgi | 2 (0.2%) |
| Perro de Presa Canario | 1 (<0.1%) |
| Pit Bull | 2 (0.2%) |
| Plott | 1 (<0.1%) |
| Pointer | 1 (<0.1%) |
| Poodle (Standard) | 4 (0.4%) |
| Rat Terrier | 1 (<0.1%) |
| Rhodesian Ridgeback | 1 (<0.1%) |
| Rottweiler | 6 (0.5%) |
| Schipperke | 3 (0.3%) |
| Schnauzer (Giant) | 4 (0.4%) |
| Shetland Sheepdog | 3 (0.3%) |
| Shiba Inu | 1 (<0.1%) |
| Shih Tzu | 1 (<0.1%) |
| Shiloh Shepherd | 1 (<0.1%) |
| Stabyhoun | 1 (<0.1%) |
| Staffordshire Bull Terrier | 3 (0.3%) |
| Vizsla | 4 (0.4%) |
| Weimaraner | 5 (0.4%) |
| Welsh Springer Spaniel | 1 (<0.1%) |
| White German Shepherd Dog | 1 (<0.1%) |
| White Swiss Shepherd Dog | 2 (0.2%) |
| Wirehaired Pointing Griffon | 2 (0.2%) |

Supplementary Table 5. Breeds/breed-types comprising >5% of the sample.

| Breed | n | percent |
| --- | --- | --- |
| Mixed breed | 70 | 6.3 |
| German Shorthaired Pointer | 85 | 7.6 |
| Cross-breed/Designer breed | 92 | 8.2 |
| Belgian Malinois | 123 | 11 |
| German Shepherd | 171 | 15.3 |
| Labrador Retriever | 214 | 19.2 |

Supplementary Table 6. Number of missing observations, mean, standard deviation, maximum, minimum, skewness, kurtosis, Shapiro-Wilk statistic, and Shapiro-Wilk p-value for each survey item.

| **Item** | **Number of Missing Items** | **Mean** | **Standard Deviation** | **Min.** | **Max.** | **Skewness** | **Kurtosis** | **Shapiro-Wilk Statistic** | **Shapiro-Wilk P-value** |
| --- | --- | --- | --- | --- | --- | --- | --- | --- | --- |
| TRAIN01 | 15 | 1.08 | 0.81 | 0 | 4 | 0.63 | 0.6 | 0.84 | 9.655306e-32 |
| TRAIN02 | 5 | 0.92 | 0.74 | 0 | 4 | 0.77 | 1.41 | 0.81 | 1.449781e-34 |
| TRAIN03 | 21 | 1.18 | 0.85 | 0 | 4 | 0.54 | 0.33 | 0.86 | 2.808713e-30 |
| TRAIN04 | 3 | 0.86 | 0.67 | 0 | 3 | 0.38 | 0.02 | 0.8 | 3.703645e-35 |
| TRAIN05 | 45 | 0.93 | 0.91 | 0 | 4 | 0.84 | 0.42 | 0.83 | 2.650988e-32 |
| TRAIN06 | 12 | 0.62 | 0.68 | 0 | 4 | 0.96 | 1.22 | 0.75 | 9.812663e-38 |
| TRAIN07 | 0 | 1.07 | 0.79 | 0 | 4 | 0.37 | -0.21 | 0.85 | 2.169221e-31 |
| TRAIN08 | 9 | 0.63 | 1.11 | 0 | 4 | 1.79 | 2.22 | 0.62 | 8.418851e-44 |
| AGG09 | 5 | 0.18 | 0.51 | 0 | 4 | 3.93 | 19.58 | 0.38 | 1.220653e-51 |
| AGG10 | 0 | 0.56 | 0.87 | 0 | 4 | 1.72 | 2.83 | 0.68 | 1.469412e-41 |
| AGG11 | 12 | 0.08 | 0.33 | 0 | 4 | 5.44 | 36.75 | 0.24 | 4.800148e-55 |
| AGG12 | 8 | 0.06 | 0.32 | 0 | 3 | 5.94 | 39.28 | 0.2 | 5.713450e-56 |
| AGG13 | 32 | 0.07 | 0.39 | 0 | 4 | 6.99 | 55.84 | 0.18 | 5.587363e-56 |
| AGG14 | 10 | 0.63 | 0.75 | 0 | 4 | 1.24 | 1.69 | 0.75 | 4.134448e-38 |
| AGG15 | 18 | 0.19 | 0.6 | 0 | 4 | 4.09 | 18.92 | 0.36 | 4.234352e-52 |
| AGG16 | 12 | 0.63 | 0.87 | 0 | 4 | 1.65 | 2.95 | 0.71 | 7.268859e-40 |
| AGG17 | 12 | 0.06 | 0.29 | 0 | 4 | 7.24 | 71.62 | 0.19 | 5.385032e-56 |
| AGG18 | 35 | 1.1 | 1.12 | 0 | 4 | 0.92 | 0.12 | 0.83 | 4.719855e-32 |
| AGG19 | 21 | 0.22 | 0.55 | 0 | 4 | 3.35 | 14.17 | 0.44 | 7.828654e-50 |
| AGG20 | 50 | 0.47 | 0.78 | 0 | 4 | 2 | 4.6 | 0.64 | 1.700955e-42 |
| FEAR21 | 17 | 0.23 | 0.56 | 0 | 4 | 3.04 | 11.04 | 0.46 | 2.166957e-49 |
| FEAR22 | 13 | 0.52 | 0.86 | 0 | 4 | 1.96 | 3.9 | 0.64 | 6.331022e-43 |
| FEAR23 | 28 | 0.23 | 0.56 | 0 | 4 | 2.96 | 10.68 | 0.47 | 7.401603e-49 |
| FEAR24 | 24 | 0.27 | 0.61 | 0 | 4 | 2.79 | 9.12 | 0.49 | 4.035360e-48 |
| FEAR25 | 10 | 0.2 | 0.53 | 0 | 4 | 3.49 | 15.47 | 0.42 | 1.678558e-50 |
| FEAR26 | 17 | 0.62 | 0.88 | 0 | 4 | 1.57 | 2.35 | 0.71 | 7.574245e-40 |
| FEAR27 | 29 | 0.52 | 0.74 | 0 | 4 | 1.59 | 2.83 | 0.69 | 1.294666e-40 |
| FEAR28 | 9 | 0.35 | 0.6 | 0 | 4 | 1.89 | 4.41 | 0.61 | 2.215563e-44 |
| FEAR29 | 19 | 0.66 | 0.82 | 0 | 4 | 1.36 | 2.07 | 0.75 | 7.562087e-38 |
| FEAR30 | 13 | 0.37 | 0.63 | 0 | 4 | 2.05 | 5.84 | 0.61 | 2.493507e-44 |
| FEAR31 | 53 | 0.79 | 0.89 | 0 | 4 | 1.04 | 0.66 | 0.79 | 1.030109e-34 |
| SEPR32 | 16 | 0.62 | 0.94 | 0 | 4 | 1.51 | 1.64 | 0.7 | 1.456687e-40 |
| SEPR33 | 14 | 0.84 | 0.96 | 0 | 4 | 1 | 0.4 | 0.8 | 4.779081e-35 |
| SEPR34 | 16 | 0.29 | 0.65 | 0 | 4 | 2.51 | 6.53 | 0.51 | 6.895010e-48 |
| EXCITE35 | 17 | 2.04 | 1.06 | 0 | 4 | -0.2 | -0.68 | 0.91 | 2.640374e-25 |
| EXCITE36 | 17 | 2.2 | 0.98 | 0 | 4 | -0.47 | -0.32 | 0.88 | 5.274969e-28 |
| EXCITE37 | 22 | 1.92 | 1.05 | 0 | 4 | 0.03 | -0.73 | 0.91 | 7.649087e-25 |
| EXCITE38 | 21 | 2.41 | 1.02 | 0 | 4 | -0.52 | -0.27 | 0.89 | 1.090840e-27 |
| ATT39 | 21 | 3.15 | 1 | 0 | 4 | -1.06 | 0.41 | 0.79 | 2.958991e-35 |
| ATT40 | 23 | 0.7 | 0.9 | 0 | 4 | 1.43 | 2.01 | 0.75 | 6.071451e-38 |
| ATT41 | 21 | 1.73 | 1.15 | 0 | 4 | 0.17 | -0.69 | 0.91 | 8.328397e-25 |
| ATT42 | 29 | 1.08 | 1.06 | 0 | 4 | 0.75 | -0.13 | 0.84 | 2.496265e-31 |
| PLAY43 | 38 | 2.96 | 1.13 | 0 | 4 | -1.02 | 0.26 | 0.81 | 1.858605e-33 |
| PLAY44 | 28 | 3.17 | 1.19 | 0 | 4 | -1.31 | 0.62 | 0.72 | 2.666095e-39 |
| PLAY45 | 32 | 3.18 | 1.12 | 0 | 4 | -1.32 | 0.85 | 0.74 | 3.087604e-38 |
| PLAY46 | 36 | 3.38 | 1.02 | 0 | 4 | -1.79 | 2.5 | 0.65 | 2.963994e-42 |
| IMP47 | 32 | 1.42 | 0.88 | 0 | 4 | 0.32 | -0.07 | 0.88 | 4.726709e-28 |
| IMP48 | 33 | 1.04 | 0.82 | 0 | 4 | 0.66 | 0.52 | 0.84 | 1.375901e-31 |
| IMP49 | 31 | 1.46 | 0.93 | 0 | 4 | 0.5 | 0.08 | 0.88 | 5.181349e-28 |
| MISC50 | 448 | 0.79 | 1.06 | 0 | 4 | 1.2 | 0.4 | 0.74 | 6.079695e-31 |
| MISC51 | 67 | 0.54 | 0.91 | 0 | 4 | 1.84 | 3.04 | 0.65 | 5.828757e-42 |
| MISC52 | 64 | 1.21 | 1.17 | 0 | 4 | 0.77 | -0.28 | 0.85 | 2.831922e-30 |
| MISC53 | 66 | 0.79 | 1.02 | 0 | 4 | 1.27 | 1.01 | 0.76 | 8.306130e-37 |
| MISC54 | 39 | 0.56 | 0.78 | 0 | 4 | 1.36 | 1.63 | 0.72 | 2.609030e-39 |
| MISC55 | 41 | 0.25 | 0.62 | 0 | 4 | 2.98 | 10.37 | 0.46 | 8.496991e-49 |
| MISC56 | 41 | 0.39 | 0.7 | 0 | 4 | 2 | 4.16 | 0.6 | 3.149371e-44 |
| MISC57 | 41 | 1.57 | 1.05 | 0 | 4 | 0.28 | -0.46 | 0.9 | 2.073455e-25 |
| MISC58 | 46 | 0.17 | 0.52 | 0 | 4 | 3.83 | 17.35 | 0.37 | 2.217191e-51 |
| MISC59 | 37 | 0.83 | 0.92 | 0 | 4 | 0.97 | 0.53 | 0.8 | 2.528656e-34 |
| MISC60 | 40 | 2.39 | 1.01 | 0 | 4 | -0.27 | -0.28 | 0.9 | 1.086574e-25 |
| MISC61 | 40 | 2.78 | 0.97 | 0 | 4 | -0.53 | -0.22 | 0.88 | 2.092420e-28 |
| MISC62 | 41 | 1.42 | 1.08 | 0 | 4 | 0.58 | -0.21 | 0.89 | 2.327415e-27 |
| MISC63 | 42 | 1.09 | 0.95 | 0 | 4 | 0.73 | 0.26 | 0.86 | 2.651403e-30 |
| MISC64 | 51 | 0.99 | 0.85 | 0 | 4 | 0.94 | 1.3 | 0.82 | 1.073411e-32 |
| MISC65 | 38 | 0.97 | 0.75 | 0 | 4 | 0.65 | 0.95 | 0.82 | 4.054022e-33 |
| MISC66 | 40 | 0.56 | 0.68 | 0 | 4 | 1.11 | 1.41 | 0.73 | 1.950999e-38 |
| MISC67 | 39 | 0.46 | 0.62 | 0 | 4 | 1.31 | 2.15 | 0.68 | 9.185168e-41 |
| MISC68 | 39 | 0.12 | 0.44 | 0 | 4 | 4.64 | 24.9 | 0.29 | 2.132719e-53 |
| MISC69 | 50 | 0.17 | 0.61 | 0 | 4 | 4.14 | 18.06 | 0.31 | 1.166681e-52 |
| MISC70 | 38 | 1.05 | 1.08 | 0 | 4 | 0.83 | -0.06 | 0.84 | 5.926984e-32 |
| MISC71 | 40 | 0.52 | 0.88 | 0 | 4 | 1.85 | 3.1 | 0.64 | 9.553892e-43 |

**
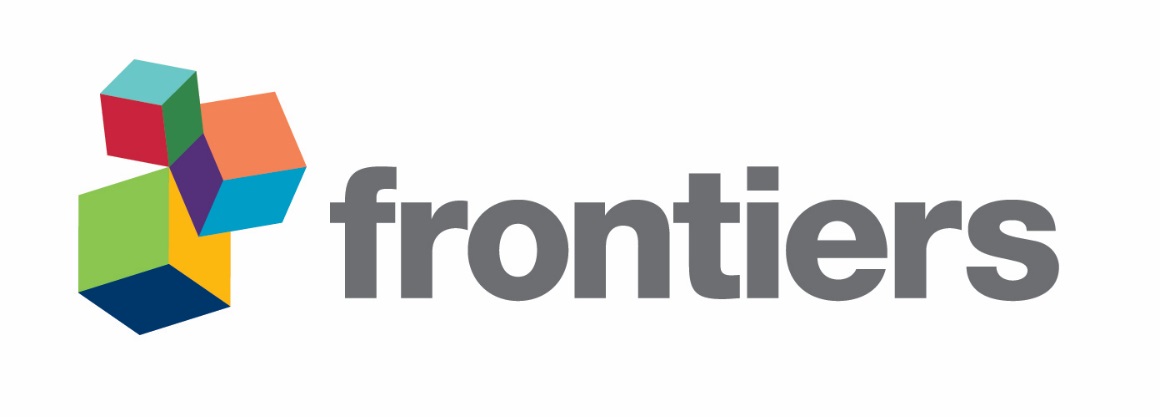
**
